# Ablation of Potassium-Chloride Cotransporter Type 3 (*Kcc3*) in Mouse Causes Multiple Cardiovascular Defects and Isosmotic Polyuria

**DOI:** 10.1371/journal.pone.0154398

**Published:** 2016-05-11

**Authors:** Alexandre P. Garneau, Andrée-Anne Marcoux, Micheline Noël, Rachelle Frenette-Cotton, Marie-Claude Drolet, Jacques Couet, Richard Larivière, Paul Isenring

**Affiliations:** 1 Nephrology Research Group, Centre de recherche L’Hôtel-Dieu de Québec, Centre hospitalier universitaire de Québec, Québec City, Canada; 2 Valvulopathy Research Group, Centre de recherche de l’Institut universitaire de cardiologie et de pneumologie de Québec, Québec City, Canada; 3 Department of Medicine, Université Laval, Québec City, Canada; University of Bern, SWITZERLAND

## Abstract

Inactivation of *Kcc3* in a mixed 129/Sv×C57BL/6 mouse background has been previously found to increase systemic blood pressure (BP) through presumed neurogenic mechanisms. Yet, while this background is generally not considered ideal to investigate the cardiovascular system, KCC3 is also expressed in the arterial wall and proximal nephron. In the current study, the effects of *Kcc3* ablation was investigated in a pure rather than mixed C57BL/6J background under regular- and high-salt diets to determine whether they could be mediated through vasculogenic and nephrogenic mechanisms. Aortas were also assessed for reactivity to pharmacological agents while isolated from the influence of sympathetic ganglia. This approach led to the identification of unforeseen abnormalities such as lower pulse pressure, heart rate, aortic reactivity and aortic wall thickness, but higher diastolic BP, left ventricular mass and urinary output in the absence of increased catecholamine levels. Salt loading also led systolic BP to be higher, but to no further changes in hemodynamic parameters. Importantly, aortic vascular smooth muscle cells and cardiomyocytes were both found to express KCC3 abundantly in heterozygous mice. Hence, *Kcc3* inactivation in our model caused systemic vascular resistance and ventricular mass to increase while preventing extracellular fluid volume to accumulate. Given that it also affected the physiological properties of aortas *in vitro*, vasculogenic mechanisms could therefore account for a number of the hemodynamic abnormalities observed.

## Introduction

The cation-Cl^−^ cotransporter (CCC) family (or *Slc12a* family) is comprised of nine members that are highly homologous to each other and that play important physiological roles. It is comprised of four closely related phylogenetic branches [1]. One of these branches includes the Na^+^-coupled cotransporters that promote net Cl^−^ entry into cells, i.e., of the Na^+^-K^+^-Cl^−^ cotransporters (NKCC1 and NKCC2) and the Na^+^-Cl^−^ cotransporter (NCC). Another branch includes the Na^+^-independent cotransporters that promote net Cl^−^ exit from cells, i.e., of the K^+^-Cl^−^ cotransporters (KCC1, KCC2, KCC3 and KCC4).

There is strong evidence to suggest that several CCC family members are involved in blood pressure (BP) regulation. This is the case for NKCC2 [2–4] and NCC [3–7] that both play a role in extracellular fluid volume (ECFV) maintenance. In particular, these carriers have been genetically linked to abnormal BP in mouse and human through mutations or polymorphisms. This is also the case for NKCC1, but the mechanisms involved differ. Indeed, inactivation of NKCC1 in mouse causes systemic hypotension [8] and impaired cardiac contractility [9] while arterial blood vessels isolated from rodents exhibit lower responsiveness to vasopressor agents under conditions where NKCC1 activity is reduced [8,10,11]. Interestingly, mutations or polymorphisms in with-no-lysine kinases (WNKs) and in STE20/SPS1-related proline/alanine-rich kinase (SPAK), both of which regulate the activity of many CCCs, have also been linked to abnormal BP [12–17].

Until recently, CCCs involved in BP regulation have included the Na^+^-coupled cotransporters exclusively. There is now evidence to suggest that the Na^+^-independent cotransporters are also involved. In three different studies, for instance, mice inactivated for *Kcc3* (or *Slc12a6*), as in the *Kcc3*^−/−^_129/Sv×C57BL/6_ model, have been found to exhibit higher mean arterial pressure (MAP) than wild-type (WT) littermates [18–20]. In one of these studies, interestingly, Rust et al. [20] have also found that *Kcc3*^−/−^_129/Sv×C57BL/6_ mice exhibited higher levels of urinary catecholamines, greater BP sensitivity to pharmacological inhibition by cholinergic or adrenergic receptor blockers, and a variety of neurological abnormalities. Accordingly, they concluded that high BP developed in the mutant animals through neurogenic mechanisms. Of notice, *Kcc3* has also been linked to corpus callosum agenesis and progressive sensorimotor neuropathy in human [21].

It is noteworthy that in the previously exploited 129/Sv×C57BL/6 background, *Kcc3*^−/−^ inactivation was also characterized by hypertrophied, Cl^−^-rich saphenous arterial vascular smooth muscle cells (VSMCs) and by increased plasma aldosterone levels in the absence of renin suppression [20]. Hence, other mechanisms in the development of high BP could have been at cause, such as suppressed K^+^-Cl^−^ cotransport in VSMCs, implying that KCC3 and NKCC1 would play reciprocal functional roles in this cell type, consistent with their reciprocal transport roles. To this effect, a number of CCCs can affect cell growth, *Kcc3* is expressed in VSMCs [20,22] and NKCC1 inhibition in isolated arterial vessels decreases vessel wall Cl^−^ content [23]. It should be noted, lastly, that the effect of dietary challenges in *Kcc3*^−/−^_129/Sv×C57BL/6_ mice was not reported, nor was the effect of *Kcc3* inactivation in the genetic backgrounds typically used to study cardiovascular or renal function in animal models.

In this study, we have analyzed a murine model of gene disruption in a purified C57BL/6J strain to revisit the role of KCC3 in the cardiovascular tissue. Compared to WT littermates, *Kcc3*^−/−^ mice exhibited higher diastolic BP (DBP), lower pulse pressure (PP), decreased aortic reactivity to adrenergic stimulation, enlarged left ventricular mass and isosmotic polyuria, but normal levels of circulating catecholamines. Taken together, our findings point towards the involvement of vasculogenic mechanisms in the development of hemodynamic disorders due to *Kcc3* inactivation and a potential role for K^+^-Cl^−^ cotransport in cardiac growth.

## Material and Methods

### Source of supplies or equipment

- Mice:
  - *Kcc3*^+/−^_129/SvEvBrd×C57BL/6J_: Mutant Mouse Regional Resource Centers, University of Carolina, Chapel Hill, NC.
  - *Kcc3*^+/+^_C57BL/6J:_ The Jackson Laboratory, Bar Harbor, ME, USA.
- Metabolic cages: Nalgene, Missisauga, ON, Canada.
- Echocardiographic system: Philips Medical Imaging, Andover, MA, USA.
- BP-series: Visitech Systems, Inc., Apex, NC, USA.
- Aortic reactivity:
  - Tungsten wires and fluid chambers: Radnoti, LLC, Monrovia, CA (catalog numbers: 158816-005T and 158817-005T).
  - Reagents: Sigma-Aldrich, Inc., St. Louis, MO, USA.
- Hematological parameters: VetScan HM5 hardware, Abaxis, Inc., Union City, CA, USA.
- Antibody-based kits for hormone measurements:
  - Aldosterone: CUSABIO^®^, Wuhan, China.
  - Epinephrine and renin: Uscn Life Science, Inc., Wuhan, China.
  - Norepinephrine: BMassay, Beijing, China.
  - Cortisol: Cobas^®^/Roche Molecular Diagnostics, Pleasanton, USA.
- Superfrost® Plus glass slides: VWR International, Radnor, PA.
- Quantitative PCR:
  - RNeasy protocol kit: Qiagen, Venlo, Limbourg, Netherlands.
  - cDNA synthesis: SuperScript™ III First-Strand Synthesis System kit for RT-PCR: Invitrogen; Life Technologies, Carlsbrad, CA, USA.
  - PCR: Platinum^®^ SYBR^®^ Green qPCR SuperMix-UDG kit: Invitrogen; Life Technologies, Carlsbrad, CA, USA.
  - cDNA synthesis and PCR: qScript™ cDNA SuperMix and PerfeCTa® SYBR® Green FastMix® Reaction Mixes, VWR International, Radnor, PA, USA.
  - Thermocycler: Stratagene Mx3005P, Agilent Technologies, Inc., Santa Clara, CA, USA.
  - Analysis: Stratagene MxPro software, Agilent Technologies, Inc., Santa Clara, CA, USA.

### Animals

All studies were approved by Laval University under animal protocol 11–029. Animals were housed at the L’Hôtel-Dieu de Québec animal facility. *Kcc3*^−/−^_C57BL/6J_ mice were generated by breeding *Kcc3*^+/−^_129/SvEvBrd×C57BL/6J_ mice in the C57BL/6J background. The mutant allele contains a gene trap called Gt(IRESBetageo)105Lex that prevents gene translation beyond exon 2 and that codes for β-galactosidase. In each experiment, animals used were males, age-matched (14 to 26 weeks old) and from the same generation (F_5_ to F_8_). When necessary, anesthesia was induced through isoflurane inhalation. Euthanasia was carried out through cervical dislocation or cardiac exsanguination.

### Hemodynamic measurements

BP and heart rate were measured by tail cuff using the BP-2000 Series II hardware system. Cuffs were 6.35 mm in diameter and platforms set at 34°C. For each experiment, 27 measurements per mouse were obtained every day (unless mentioned otherwise) between 13:00 and 15:00 in appropriately-sized holders during 10 days. The following values were not included in the analyses: those obtained during an acclimation period (measurements of the first 6 days and first 8 measurements of the 4 remaining days) as well as outliers (defined as values [V] < [Q_1_−1.5 **×** IQR] or > [Q_3_ + 1.5 × IQR], where Q stands for quartile and IQR for interquartile range). MAP and PP were calculated from systolic BP (SBP) and DBP.

**Fig 1:**
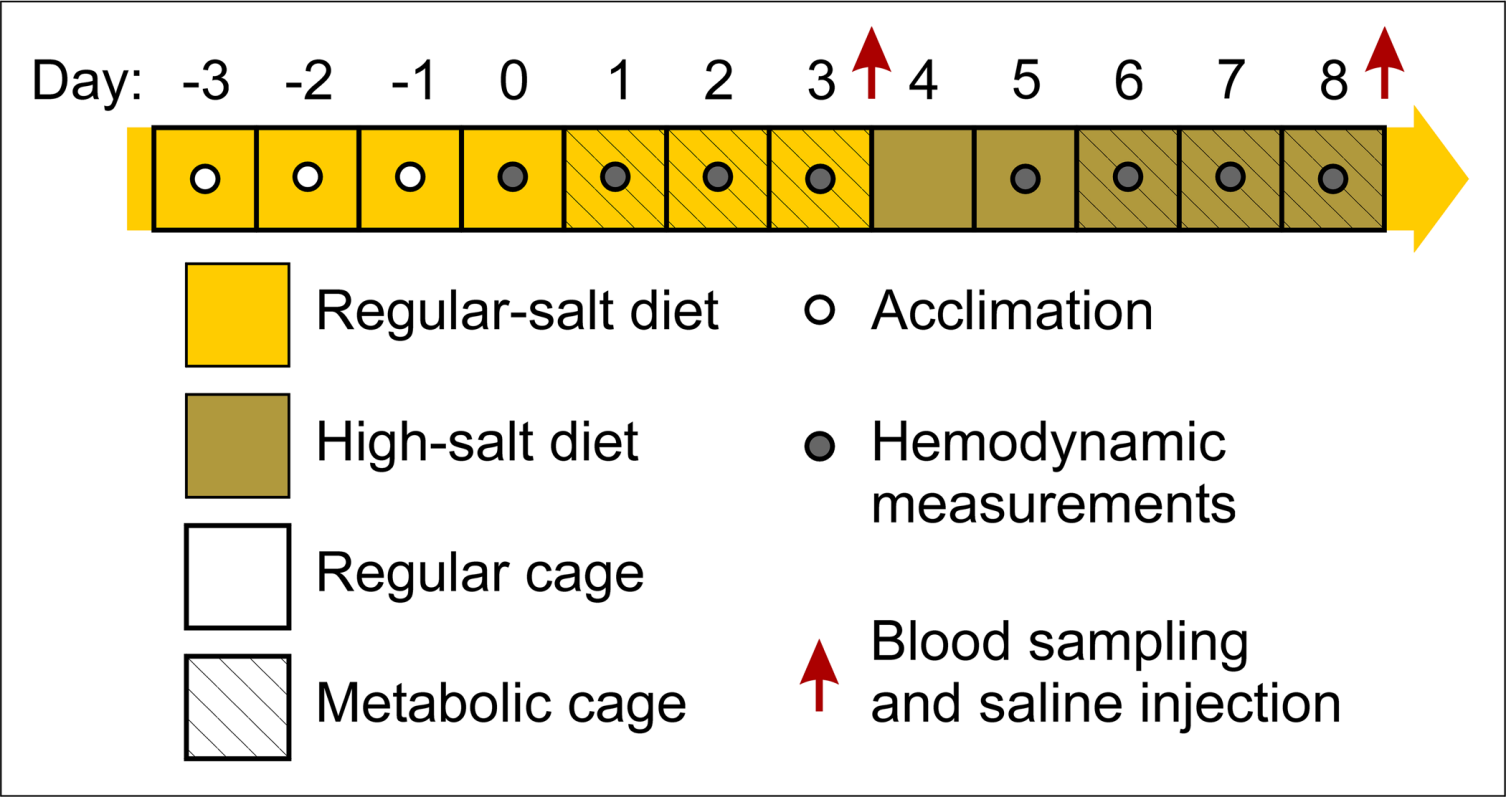
Na^+^ loading protocol. Mice were subjected to 7 days of regular diet (0.26% NaCl, ~10 mg/d) followed by 5 days of high-salt diet (8% NaCl, ~300 mg/d). Days -3 to -1 served as an acclimation period. BP and HR measurements (23 per day) were obtained between 16:30 and 18:45. Those obtained during acclimation, the first 8 daily hemodynamic measurements and the outliers (as defined in *Material and Methods*) were excluded from further analyses. Chow and water intake, diuresis, fecal output and biochemical parameters were measured at different time points in Nalgene diuresis cages.

In some experiments, mice were fed a high-salt diet in metabolic cages to determine whether the effect of *Kcc3* inactivation could be amplified through changes in ECFV. A schematic representation of the protocol used for Na^+^ loading is described in Fig 1. Briefly, mice were subjected to 7 days of regular diet (0.26% NaCl, ~10 mg/d) followed by 5 days of high-salt diet (8% NaCl, ~300 mg/d) while chow and water intake, diuresis, feces output, BP, heart rate and biochemical parameters were measured at different time points.

### Aortic reactivity

Isolated thoracic aortas freed of adipose tissue were maintained in ice-cold 25 mmol L^-1^ HEPES-buffered Krebs-Henseleit solution and cut transversally into 2-mm segments (4 segments per aorta). Subsequently, each of the isolated segments was threaded through its lumen by two 0.127-mm tungsten wires and placed into a chamber filled with 95% O_2_/5% CO_2_-aerated bicarbonate-buffered Krebs-Henseleit solution (called regular buffer hereafter) warmed at 38°C. One of the wires was used to immobilize the aortic segment and the other to measure isometric force development through a height-adjustable transducer.

Once mounted, each aortic segment was loaded to 9.8 mN over 45 min, primed twice with 1 μmol L^-1^ phenylephrine hydrochloride (PhE) and washed four times over 15 min in regular buffer until the preload contractile state was recovered. Subsequently, dose-response curves were generated by exposing aortic segments sequentially to cumulative concentrations of PhE, carbamoylcholine chloride (CCh) or sodium nitroprusside (SNP). Before assessing the effects of CCh and SNP, however, aortic segments were stably precontracted to ~12 mN using ~1 μmol L^-1^ PhE (each segment receiving a different dose depending on its initial contractile response) and after each contraction or decontraction assay, they were washed four times over 15 min in regular buffer. In one series of experiments, the effect of 1 mmol L^-1^ furosemide on precontracted aortic segments was also examined. This drug is a loop diuretic that inhibits both the NKCCs and KCCs *in vitro* [1,8,10,11,24].

### Echocardiography

A motion mode, two-dimensional and Doppler echocardiogram was performed under anesthesia using a Sonos 5500 echographic system equipped with a 12 MHz probe. Left ventricular dimensions, wall thickness, ejection fraction and cardiac output were assessed as previously reported [25,26].

### Sample preparation

Tissues and fluids were collected under anesthesia or in awakened mice. They included urine collected through different means, whole blood obtained by cardiac exsanguination, hearts, thoracic aortas, gonadal adipose depots and adrenal glands.

### Blood, serum, plasma and urine measurements

Hematological parameters were obtained in whole blood with the VetScan HM5 hardware. Urea, creatinine, electrolyte and bicarbonate concentrations were measured in serum or urine through standard procedures. Aldosterone, epinephrine, norepinephrine, renin and cortisol concentrations were measured in plasma or urine through commercially available antibody-based kits as per the manufacturers’ specifications.

### Microscopic studies

Five- to 15-μm tissue sections mounted onto Superfrost® Plus glass slides and embedded in paraffin were analyzed by haematoxylin-eosin staining. In certain experiments, they were pre-embedded in Optimal Cutting Temperature compound (OCT) for β-galactosidase activity assays through the following incubation steps: 1) 5 min in 0.1 mol L^-1^ PBS, 2 mmol L^-1^ MgCl_2_ and 5 mmol L^-1^ EGTA (called solution L), 2) 5 min in 4% paraformaldehyde and 0.2% glutaraldehyde diluted in solution L, 3) 15 min in solution L to remove the fixating agents, 4) 18 h in 0.01% Na-deoxycholate, 0.02% NP-40, 5 mmol L^-1^ K-ferricyanide, 5 mmol L^-1^ K-ferrocyanide, 2 mmol L^-1^ MgCl_2_ and 0.1 mmol L^-1^ PBS (called permeabilization solution), 5) 16 h in 0.1% X-Gal diluted in permeabilization solution, 6) 15 min in solution L, 7) 2 min in water to rinse-off solution L, 8) 5 min in nuclear fast red solution and 9) several minutes in dehydrating solutions. All slides were mounted with coverslips once the incubation steps were completed.

Histomorphometric analyses of thoracic aortas were also carried out by measuring wall thickness on transversal sections at 8 equidistant radial points. Data were calculated as means of 16 lengths per mice from 2 contiguous aortic sections among 7–8 mice per group.

### Quantitative PCR (qPCR)

Total RNA purified from homogenized tissues was amplified through qPCR using SYBR^®^ Green and gene-specific primers (see Table 1). N-fold changes in aortic gene expression were calculated as relative copy numbers using the house keeping gene hypoxanthine-guanine phosphoribosyl-transferase for normalization. N-fold changes in cardiac gene expression was calculated according to the −2ΔΔCt method as previously reported using the house keeping gene cyclophilin A for normalization [27].

**Table 1:** Oligonucleotides used for qPCR studies.

Genes	Sense	Primers
A) Aorta		
* ‒ Hprt*	forward	TGCTCGAGATGTCATGAAGG
	reverse	AATGTAATCCAGGTCAGC
* ‒ Slc12a2* (NKCC1)	forward	AGGTTCTCCAAACTCACGGCCTG
	reverse	GACCAAGTCCAGCAGCCTGCATC
B) Heart		
* ‒ Nppa* (ANP)	forward & reverse	Cat. no.: QT00366170
* ‒ Nppb* (BNP)	forward & reverse	Cat. no.: QT00183225
* ‒ Ppia* (CycA)	forward & reverse	Cat. no.: QT00177394
* ‒ Col1a1* (proCol1a1)	forward & reverse	Cat. no.: QT00370622
* ‒ Col3a1* (proCol3a1)	forward & reverse	Cat. no.: QT01083537
* ‒* Myh6 (α-MHC)	forward & reverse	Cat. no.: QT00190267
* ‒* Myh7 (β-MHC)	forward & reverse	Cat. no.: QT00189504

Sequences are written 5’ to 3’. Oligonucleotides used for amplification of heart genes were purchased from Qiagen. ANP, atrial natriuretic peptide; BNP, B-type natriuretic factor; Cat. no., catalogue number; CycA, cyclophilin A; Hprt, hypoxanthine-guanine phosphoribosyltransferase.

### Wire hang test

A number of studies were carried out to determine how allele expression in the C57BL/6J background compared with allele expression in the genetic backgrounds used in previous studies. They included the wire hang test to assess balance and prehensile strength. For this procedure, mice were placed onto a 10-cm square grid that was flipped upside down 50 cm over a cushioned surface and time to fall off the grid was measured.

### Statistics

Values are given as mean ± SEM using *n* to indicate the number of animals or specimens. Data were compared using parametric as well as non-parametric tests depending on sample size and distribution type. Differences were considered significant at *p* < 0.05.

## Results

### Hemodynamic parameters

**Fig 2:**
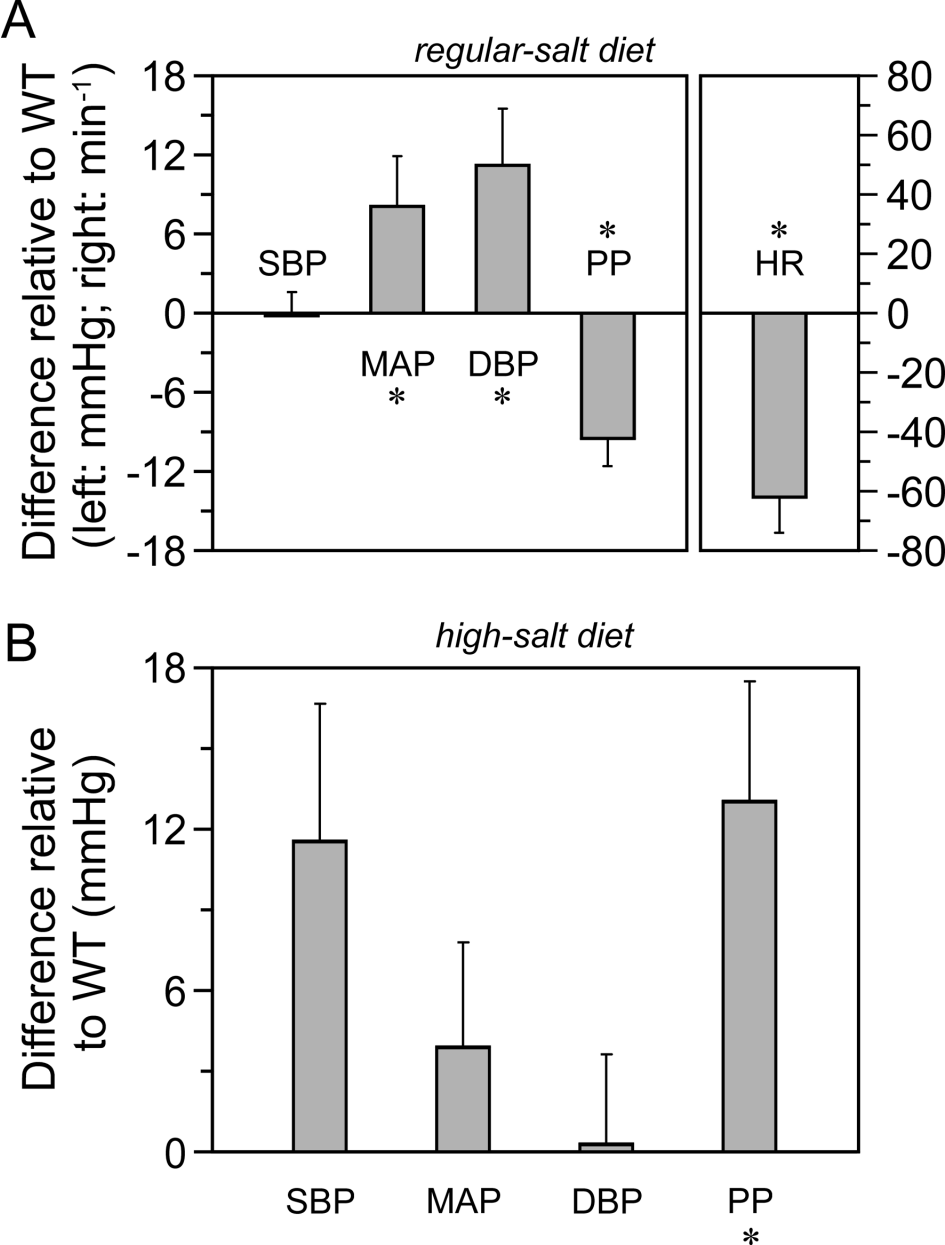
Hemodynamic parameters. Measurements were obtained by tail cuff sphygmomanometry using the BP-2000 Series II system. Data are presented as means of daily differences ± SEM between *Kcc3*^−/−^ and *Kcc3*^+/+^ mice among 4 consecutive days and 4 to 6 animals per group. **A,** Regular-salt diet. **B,** High-salt diet. The protocol used is illustrated in Fig 1. * indicates that the data are significantly different statistically from 0 (*p* < 0.05) based on a Wilcoxon rank-sum test. DBP, diastolic blood pressure; HR, heart rate; MAP, mean arterial pressure; PP, pulse pressure; SBP, systolic blood pressure. Table 2 is used to show the original data from which differences in parameters were calculated under the regular diet.

To determine whether BP in the pure *Kcc3*^−/−^_C57BL/6J_ mouse model was elevated and salt-sensitive as in many models of systemic hypertension, hemodynamic measurements were obtained by tail-cuff sphygmomanometry over several days under regular- and high-salt diets. This method was preferred over radiotelemetric measurements to prevent mutant mice from developing excessive nervousness following catheter implantation. Data are shown in Fig 2 as differences in hemodynamic parameters relative to WT littermates.

**Table 2:** Hemodynamic parameters.

Parameters	*Kcc3*^−/−^	*Kcc3*^+/+^	*p*
Systolic blood pressure (mmHg)	119.2 ± 0.8	119.4 ± 2.0	0.49
Mean arterial pressure (mmHg)	96.1 ± 2.4	88.0 ± 1.7	<0.05 ^†^
Diastolic blood pressure (mmHg)	83.7 ± 2.9	72.4 ± 1.5	<0.05 ^†^
Pulse pressure (mmHg)	37.4 ± 2.4	46.9 ± 1.0	<0.05 ^†^
Heart rate (min^-1^)	604 ± 11	666 ± 6	<0.05 ^†^

Data are expressed as means ± SEM (*n* = 4).

^†^ indicates that values in a row are significantly different statistically between each other based on a Wilcoxon rank-sum test.

Under a regular-salt diet (panel A), it is seen that MAP and DBP in *Kcc3*^−/−^ mice are significantly higher than in WT littermates while SBP are similar and PP lower. These results suggest that systemic vascular resistance as well as aortic distensibility are elevated in *Kcc3*^−/−^ mice and, accordingly, that resistive and conductive vessels react differently to *Kcc3* inactivation. In panel A, it is also seen that heart rate is significantly lower, a finding that had not been reported yet.

During a 5-day high-salt diet (panel B), differences in BP between genotypes are no longer the same. Indeed, SBP and PP in *Kcc3*^−/−^ mice are ~12 mmHg higher than in WT littermates while MAP and DBP are similar. These results suggest once again that resistive and conductive vessels exhibit different behaviors in response to *Kcc3* inactivation.

### Characterization of isolated thoracic aortas

**Fig 3:**
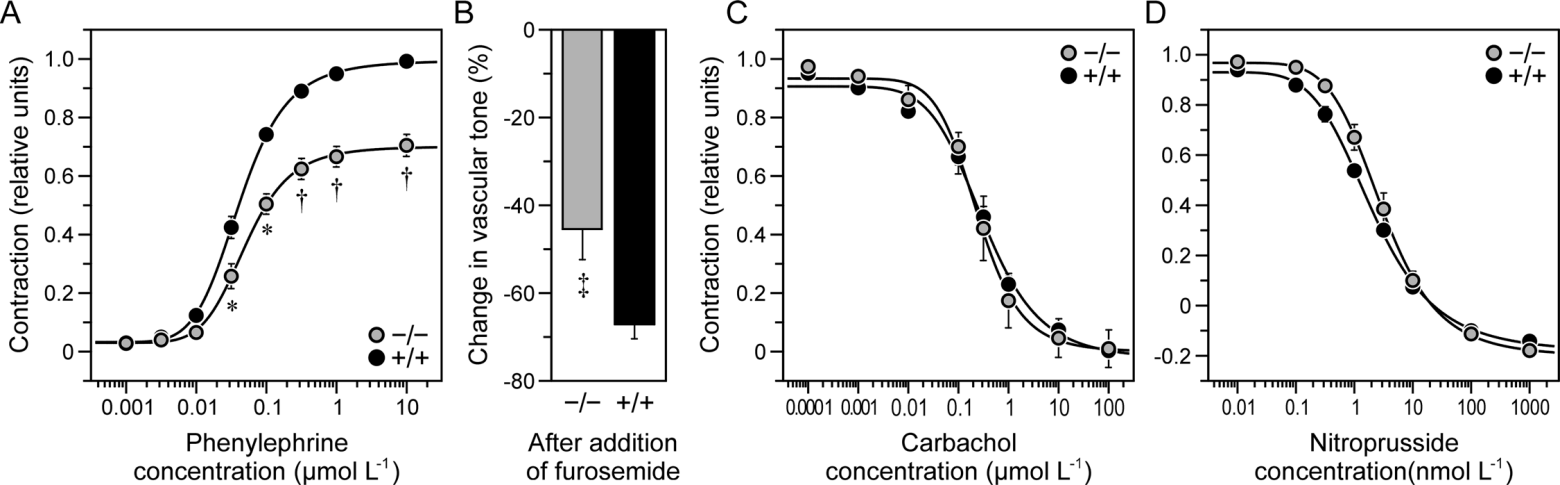
Reactivity of isolated thoracic aorta. Vessels were freed of adipose tissue, cut into 2-mm ring segments and incubated at 38°C during the assays. Differences between data sets were analyzed with the Wilcoxon rank-sum test. Except for panel A, aortas were precontracted with phenylephrine (~1 μmol L^-1^; ~12 mN contraction). **A,** Effect of phenylephrine. Data are presented as isometric force developments normalized to maximal contraction and are shown as means ± SEM of 6 mice among 6 experiments. * and † indicate that the data are significantly different statistically between *Kcc3*^−/−^ mice and wild-type littermates (*p* < 0.05 and < 0.01, respectively). **B,** Effect of furosemide (1 mmol L^-1^). Data are presented as furosemide-induced changes in the vascular tone of precontracted aortic segments and are shown as means ± SEM of 4 mice among 4 experiments. ‡ indicates that the mean is significantly different statistically compared to *Kcc3*^+/+^ littermates (*p* < 0.001). **C,** Effect of carbachol. Data are presented as isometric force development normalized to maximal contraction and are shown as means ± SEM of 5 mice among 5 experiments. **D,** Effect of nitroprusside. Data are presented as isometric force development normalized to maximal contraction and are shown as means ± SEM of 5 mice among 5 experiments.

**Fig 4:**
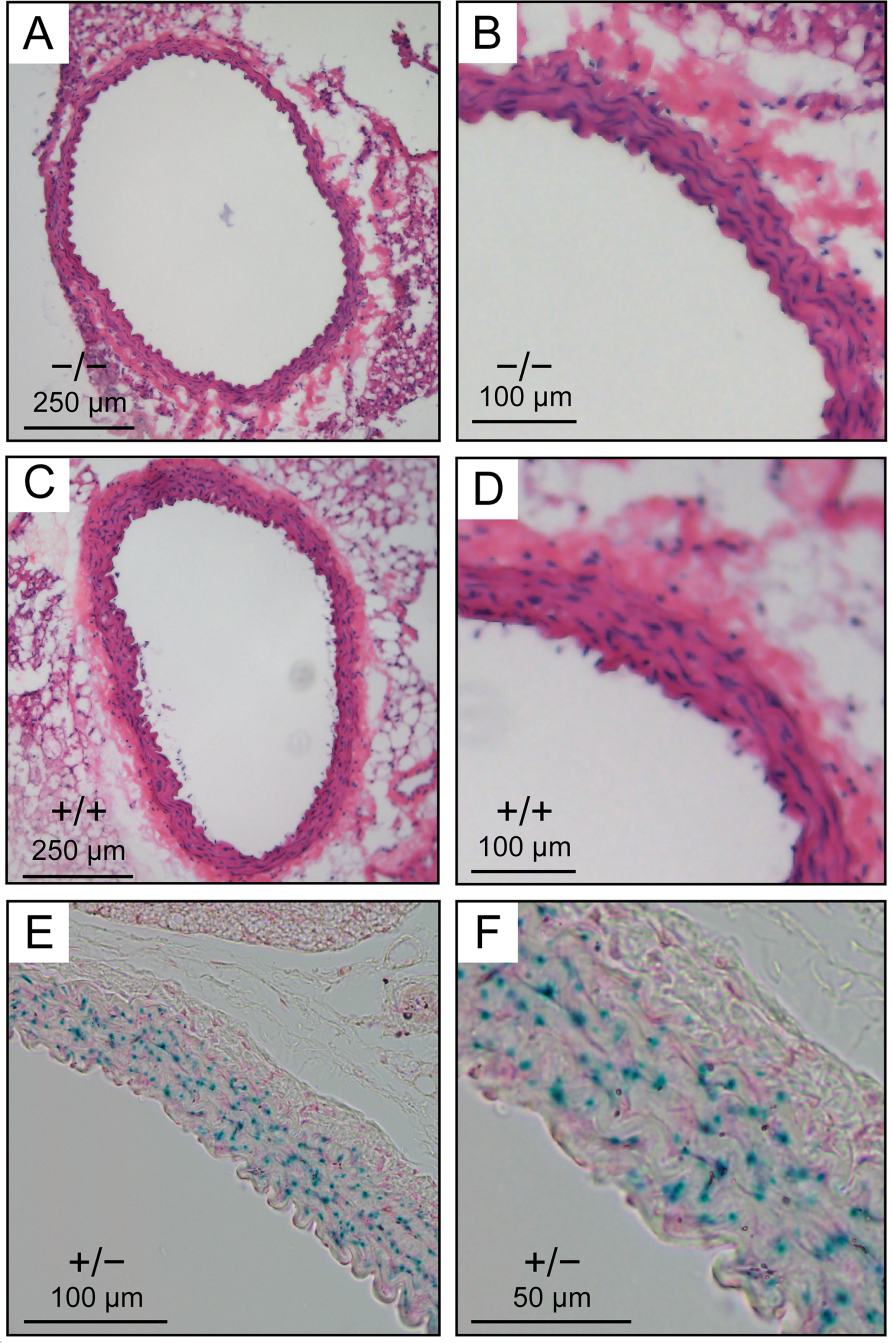
Microscopic and transcriptomal analysis of thoracic aorta. **A to D,** Histological appearance of thoracic aorta. Tissues were embedded in paraffin and stained with hematoxylin-eosin. Wall thickness was measured through histomorphometric analyses as desribed in *Material and Methods*. **E and F,** *Kcc3* expression in *Kcc3*^+/−^ thoracic aorta based on β-galactosidase activity. Enzyme detection was carried out in Optimal Cutting Temperature-embedded tissues as described in *Material and Methods*. * indicates that the mean is significantly different statistically compared to WT littermates based on a paired Student *t* test.

To determine whether lower PP in *Kcc3*^−/−^ mice was accompanied by abnormalities in conductive vessels and whether vasculogenic mechanisms could be at cause, thoracic aortas were isolated from the animals and characterized through a combination of functional, histological and expression studies while no longer under the influence of sympathetic ganglia. Results are summarized in Figs 3 and 4.

Looking first at the functional data (Fig 3), one can observe that thoracic aortas from *Kcc3*^−/−^ mice are less reactive to phenylephrine hydrochloride (PhE) than those of *Kcc3*^+/+^ littermates (panel A, gray and black circles, respectively). At saturating concentrations of PhE, the difference in force development is more than 30%. One can also observe that aortas from *Kcc3*^−/−^ mice are less sensitive to PhE in the presence of furosemide (panel B), a drug that inhibits the activity of NKCC1 and KCC3, but equally sensitive to cholinergic or nitric oxide stimulation (panels C and D). Taken together, these results indicate that decreased reactivity to adrenergic stimulation could be due to lower levels of cation-Cl^−^ cotransport in the vascular wall, but not to the absence of *Kcc3* in the endothelium or to NO-dependent mechanisms.

Looking next at the structural and expression data (Fig 4), aortic walls of *Kcc3*^−/−^ mice (panels A and B) are seen to be thinner than those of *Kcc3*^+/+^ (panels C and D) but to appear otherwise normal. Based on histomorphometric analyses, the difference in thickness was 13% (*p* = 0.0123). The mutant allele is also seen to express β-galactosidase at high levels in aortic VSMCs (panels E and F). Based on qPCR studies, lastly, *Nkcc1* expression also tended to be downregulated in *Kcc3*^−/−^ thoracic aortas (*n* = 2, data not shown). These results confirm that WT C57BL/6J mice express *Kcc3* and *Nkcc1* in the medial layer of their thoracic aorta where the two transporters would thus be functionally linked.

### Characterization of the heart

**Fig 5:**
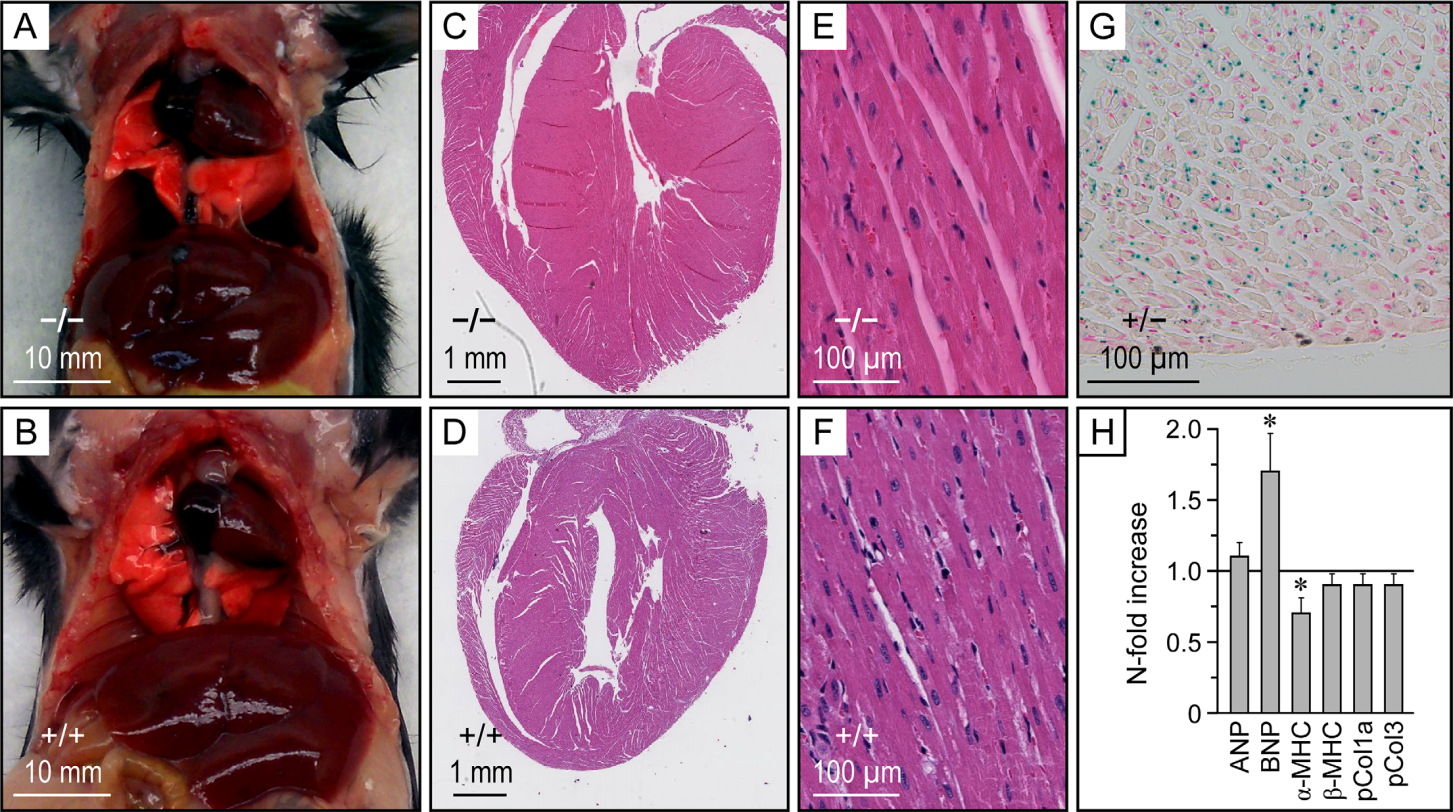
Macroscopic, microscopic and transcriptomal analysis of the heart. **A and B,** Macroscopic appearance of the thoracic cavity. Animals were 15 weeks old and from the same litter. **C to F,** Histological appearance of heart. Tissues were embedded in Optimal Cutting Temperature compound and stained with hematoxylin-eosin. **G,** *Kcc3* expression in *Kcc3*^+/−^ ventricle based on β-galactosidase activity. Enzyme detection was carried out in Optimal Cutting Temperature-embedded tissues as described in *Material and Methods*. **H,** Heart transcriptomal activity. Gene-specific primers used are listed in Table 1. Data are presented as N-fold increases in *Kcc3*^−/−^ mice relative to *Kcc3*^+/+^ mice and are shown as means ± SEM using 4–6 animals per group. * indicates that the data are significantly different statistically from 1 (*p* < 0.05) based on a Student *t* test. ANP, atrial natriuretic peptide; BNP, B-type natriuretic factor; MHC, myosin heavy chain; pCol, procollagen.

Phenotyping studies of the heart were carried out through a combination of approaches to determine whether changes in myocardial function could account for some of the observed hemodynamic abnormalities. They are presented in Fig 5 and Table 3.

**Table 3:** Cardiac function.

Parameter	*Kcc3*^−/−^	*Kcc3*^+/+^	*p*
A) Cardiac weight	(*n* = 29)	(*n* = 32)	
* *‒ Absolute (mg)	149.1 ± 2.7	141.1 ± 2.9	0.0491 *
* *‒ Relative to body weight (%)	0.510 ± 0.007	0.391 ± 0.010	< 10^−13^ *
B) Echocardiographic parameters	(*n* = 8)	(*n* = 8)	
* *‒ End-diastolic diameter (mm)	4.18 ± 0.05	3.85 ± 0.05	0.0182 ^†^
* *‒ End-systolic diameter (mm)	2.32 ± 0.10	2.24 ± 0.07	0.6988
* *‒ Ejection fraction (%)	69 ± 2	66 ± 2	0.5635
* *‒ LV weight (mg)	136 ±5	117 ± 6	0.0520
* *‒ LV weight relative to tibial length (mg mm^-1^)	6.32 ± 0.25	5.24 ± 0.30	0.0240 ^†^
* *‒ LV weight relative to body weight (%)	0.544 ± 0.025	0.408 ± 0.026	0.0054 ^†^
* *‒ Heart rate (min^-1^)	386 ± 17	423 ± 16	0.0831
* *‒ Stroke volume (μL)	61 ± 3	60 ± 4	0.9657
* *‒ Cardiac output (mL min^-1^)	24 ± 1	25 ± 1	0.3184
C) Parameters used for normalization	(*n* = 8)	(*n* = 8)	
* *‒ Body weight (g)	25.2 ± 0.8	28.8 ± 0.7	0.0054 ^†^
* *‒ Tibial length (mm)	21.61 ± 0.09	22.30 ± 0.14	0.0113 ^†^

Data are expressed as means ± SEM. Hearts weighed were freed of blood vessels and pericardial fat. The number of mice tested among several experiments is indicated between parentheses.

* and ^†^ indicate that values in a row are significantly different statistically between each other based on Student *t* and Wilcoxon rank-sum tests, respectively. LV, left ventricle.

Starting with Fig 5, one can observe that the heart of *Kcc3*^−/−^ mice (panels A and C) is enlarged macroscopically compared to WT littermates (panels B and D) while cardiac chamber size, tissue organization and cell morphology are similar between genotypes (panels C to F). One can also observe that cardiomyocytes from *Kcc3*^+/−^ mice express β-galactosidase at high levels (panel G) and that cardiomyocytes from *Kcc3*^−/−^ mice exhibit higher B-type natriuretic peptide (BNP)/α-myosin heavy chain (MHC) ratios [27] than WT littermates (panel H; fold difference was 2.4). These results suggest that cardiac mass increased through hypertrophic changes while *Kcc3* is no longer expressed in this tissue.

Continuing with Table 3, it is seen that isolated hearts are heavier in *Kcc3*^−/−^ (section A) based on absolute and normalized weight measurements (6% and 30% heavier, respectively). It is also seen that hearts in *Kcc3*^−/−^ are hypertrophied (section B) based on echocardiographic measurements, i.e., left ventricle during diastole is increased in size with larger chamber diameter (8% difference compared to WT littermates) and higher indexed left ventricular mass (25% difference). In section B, otherwise, ejection fraction, stroke volume and cardiac output are all within normal limits.

### Renal function

In the renal epithelium, *Kcc3* is expressed almost exclusively in the proximal tubule [28]. Renal function as well as water and electrolyte balance were thus assessed to determine the effect of *Kcc3* inactivation in this nephron segment specifically and on glomerular filtration secondarily. Data are shown in Table 4. As can be observed, no differences are seen in creatinine, urea, Na^+^ and H^+^ serum concentrations (section A). However, *Kcc3* inactivation is associated with higher water intake and hourly diuresis, but no difference in urinary osmolality or electrolyte concentration (sections B and C). Of notice, the difference in diuresis is almost twice the difference in water intake.

**Table 4:** Renal function.

Trait	*Kcc3*^−/−^	*Kcc3*^+/+^	*p*
A) Standard serum or blood measurements			
* *‒ Creatinine (μmol L^-1^)	13.00 ± 1.09 (7)	11.25 ± 1.46 (8)	0.5992
* *‒ Urea (mmol L^-1^)	10.77 ± 0.78 (7)	10.46 ± 0.38 (8)	0.7716
* *‒ Na^+^ (mmol L^-1^)	150.3 ± 0.6 (7)	150.5 ± 0.5 (8)	0.5022
* *‒ CO_2_ (mmol L^-1^)	14.0 ± 1.2 (7)	15.0 ± 1.2 (8)	0.2599
* *‒ Hematocrit (%)	37.2 ± 0.5 (7)	38.7 ± 1.0 (6)	0.2246
B) Water balance and food intake			
* *‒ Daily water intake (mL)	4.0 ± 0.1 (11)	3.0 ± 0.3 (9)	0.0133 ^†^
* *‒ Hourly diuresis (μL h^-1^)	50.2 ± 3.2 (11)	31.4 ± 3.6 (9)	0.0014 ^†^
* *‒ Urine osmolality (osmol kg^-1^)	1.99 ± 0.11 (5)	1.99 ± 0.15 (5)	1.000
C) Urine measurements			
* *‒ Na^+^/creatinine	34.3 ± 3.0 (7)	34.2 ± 1.8 (8)	0.955
* *‒ Creatinine (μmol L^-1^)	3.07 ± 0.12 (7)	4.78 ± 0.25 (8)	< 0.001 ^†^
* *‒ Specific gravity	1.041 ± 0.002 (7)	1.060 ± 0.003 (7)	0.002 ^†^

Studies were carried out under a regular diet. Data are expressed as means ± SEM. The number of mice tested among several experiments is indicated between parentheses.

^†^ indicates that values in a row are significantly different statistically between each other based on a Wilcoxon rank-sum test.

### Circulating factors involved in BP control

**Fig 6:**
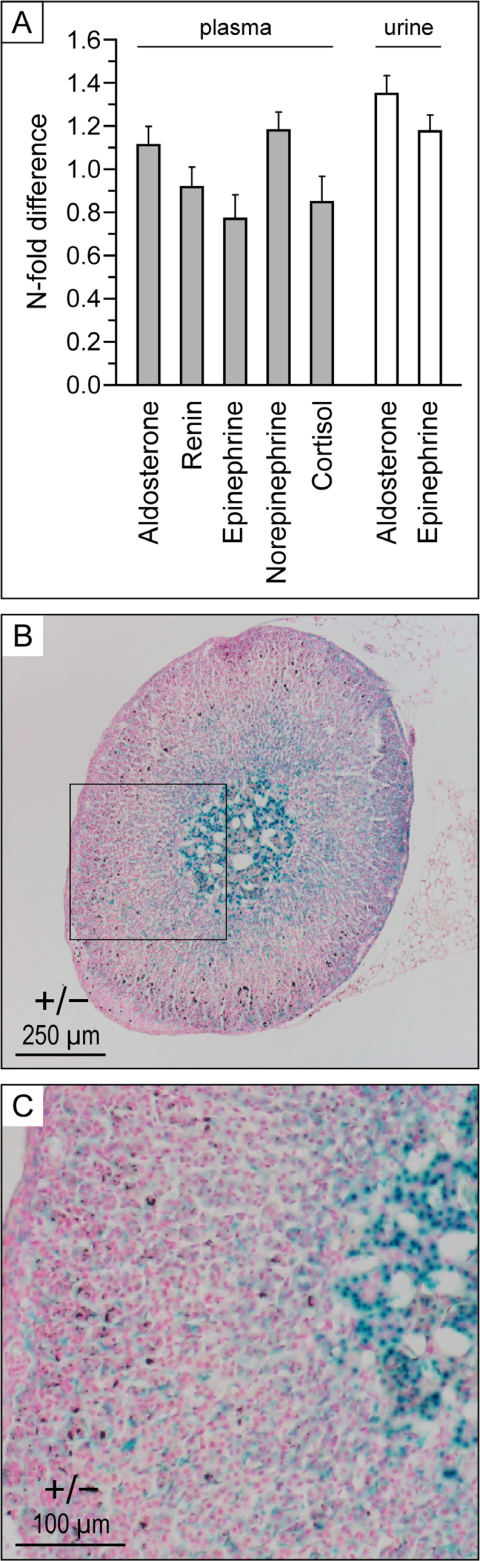
Secretion profile of hemodynamically relevant hormones. **A,** Plasma and urine measurements. Animals were under regular diets. Data are presented as N-fold changes in *Kcc3*^−/−^ mice relative to *Kcc3*^+/+^ mice and are shown as means ± SEM of 13 to 23 mice (except for cortisol where means are of 4 mice) among 1–3 experiments. None of the data shown were statistically different from 1 based on Wilcoxon rank-sum tests. Aldo, aldosterone; Epi, epinephrine; Norepi, norepinephrine. **B and C,** *Kcc3* expression in *Kcc3*^+/−^ adrenal gland based on β-galactosidase activity. Enzyme detection was carried out in Optimal Cutting Temperature-embedded tissues as described in *Material and Methods*.

Measurements of plasma or urine aldosterone, catecholamines, cortisol and renin levels were obtained under a regular diet to obtain further insight into the mechanisms of DBP elevation and cardiac hypertrophy. As shown in Fig 6, no differences are observed between *Kcc3*^−/−^ and *Kcc3*^+/+^ mice (panel A). Interestingly, *Kcc3* is detected in all four layers of the adrenal gland (panels B and C), but is more abundant in the medulla.

### Body measurements and lipid profile

**Fig 7:**
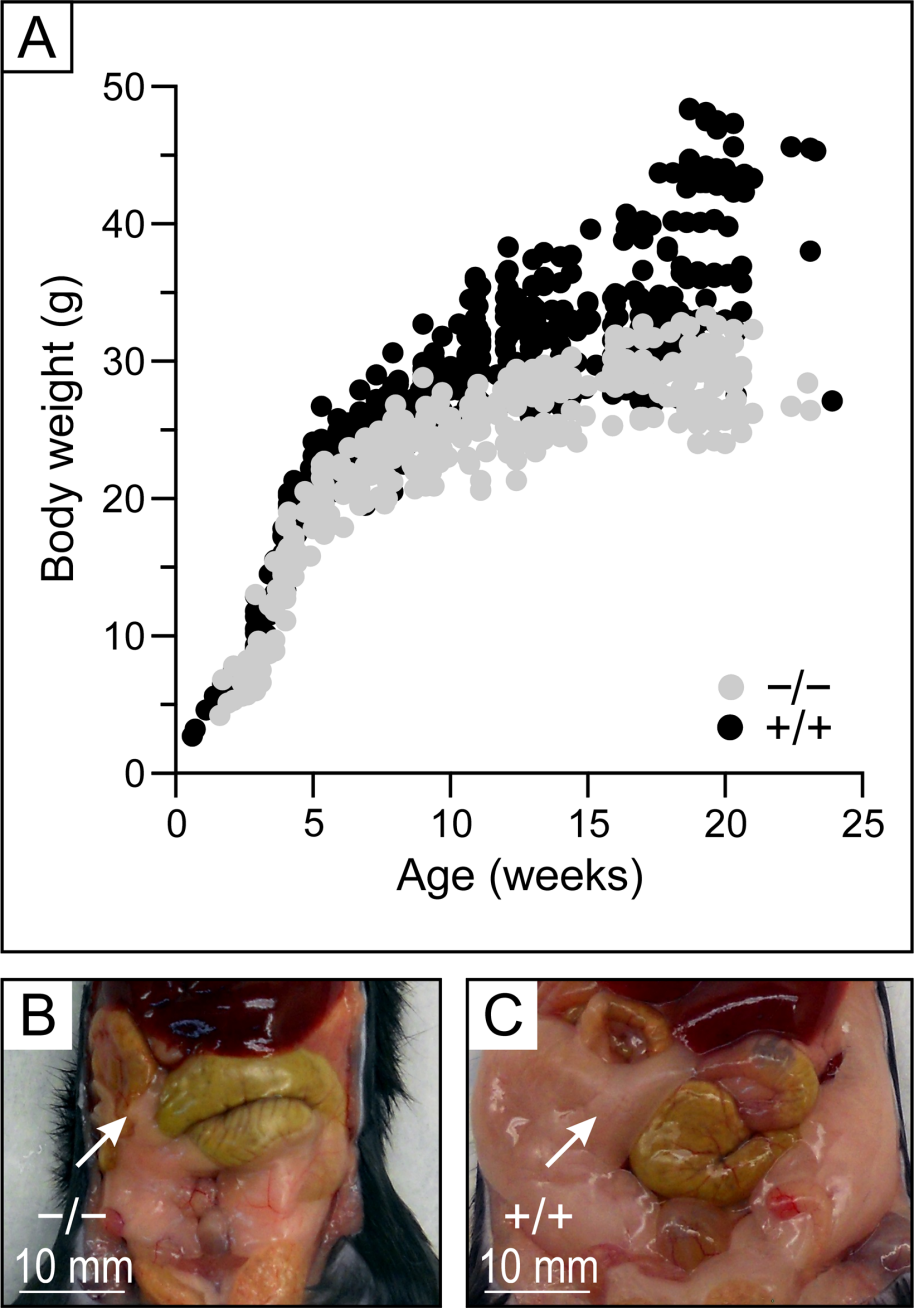
Body measurements. **A,** Growth curves. Values shown are from 57 *Kcc3*^−/−^ and 98 *Kcc3*^+/+^ animals. Additional measurements are shown in Table 3. **B and C,** Macroscopic appearance of the abdominal cavity. Animals were 15 weeks old and from the same litter. Gonadal depots (indicated by arrows) are much smaller in *Kcc3*^−/−^ mouse compared to wild-type mouse.

In addition to the cardiovascular abnormalities objectivized, it was apparent that *Kcc3* inactivation was also associated with lower body weight and substantial leanness at adult age. Additional measurements were thus carried out to quantify these other abnormalities more precisely and determine whether lower amounts of chow intake could have been at cause. They are shown in Fig 7 (panels A to C) and Table 5. Important differences are indeed observed in a number of parameters such as normalized gonadal depot weight and maximal weight based on growth curves (respectively 3.2- and 1.5-fold lower in *Kcc3*^−/−^ mice compared to *Kcc3*^+/+^ mice). Interestingly, however, chow intake is seen to be 1.7-fold higher in the mutant animals while serum cholesterol levels are seen to be lower.

**Table 5:** Body measurements and lipid profile.

Trait	*Kcc3*^−/−^	*Kcc3*^+/+^	*p*
Adult TBW (g)	29.3 ± 0.5 (29)	36.6 ± 1.1 (32)	< 10^−6^ *
GFW (g)	0.46 ± 0.09 (11)	2.07 ± 0.20 (11)	<10^−4^ ^†^
GFW relative to TBW (%)	1.51 ± 0.27 (11)	4.85 ± 0.39 (11)	0.0002 ^†^
*K*_½_ of growth curve (weeks)	3.63 (57)	6.19 (98)	n/a
*V*_max_ of growth curve (g)	30.2 (57)	44.0 (98)	n/a
Daily chow intake (g)	3.7 ± 0.1 (11)	2.2 ± 0.2 (9)	0.0005 ^†^
Cholesterol (mmol L^-1^)	1.58 ± 0.08 (7)	2.42 ± 0.11 (8)	0.0015 ^†^
Triglycerides (mmol L^-1^)	0.69 ± 0.10 (7)	0.99 ± 0.13 (8)	0.1473

Data are expressed as means ± SEM. The number of mice tested among several experiments is indicated between parentheses.

* and ^†^ indicate that values in a row are significantly different statistically between each other based on Student *t* and Wilcoxon rank-sum tests, respectively, and n/a indicate not available or not applicable. GFW, gonadal fat weight; TBW, total body weight.

### Neurologic assessment

**Fig 8:**
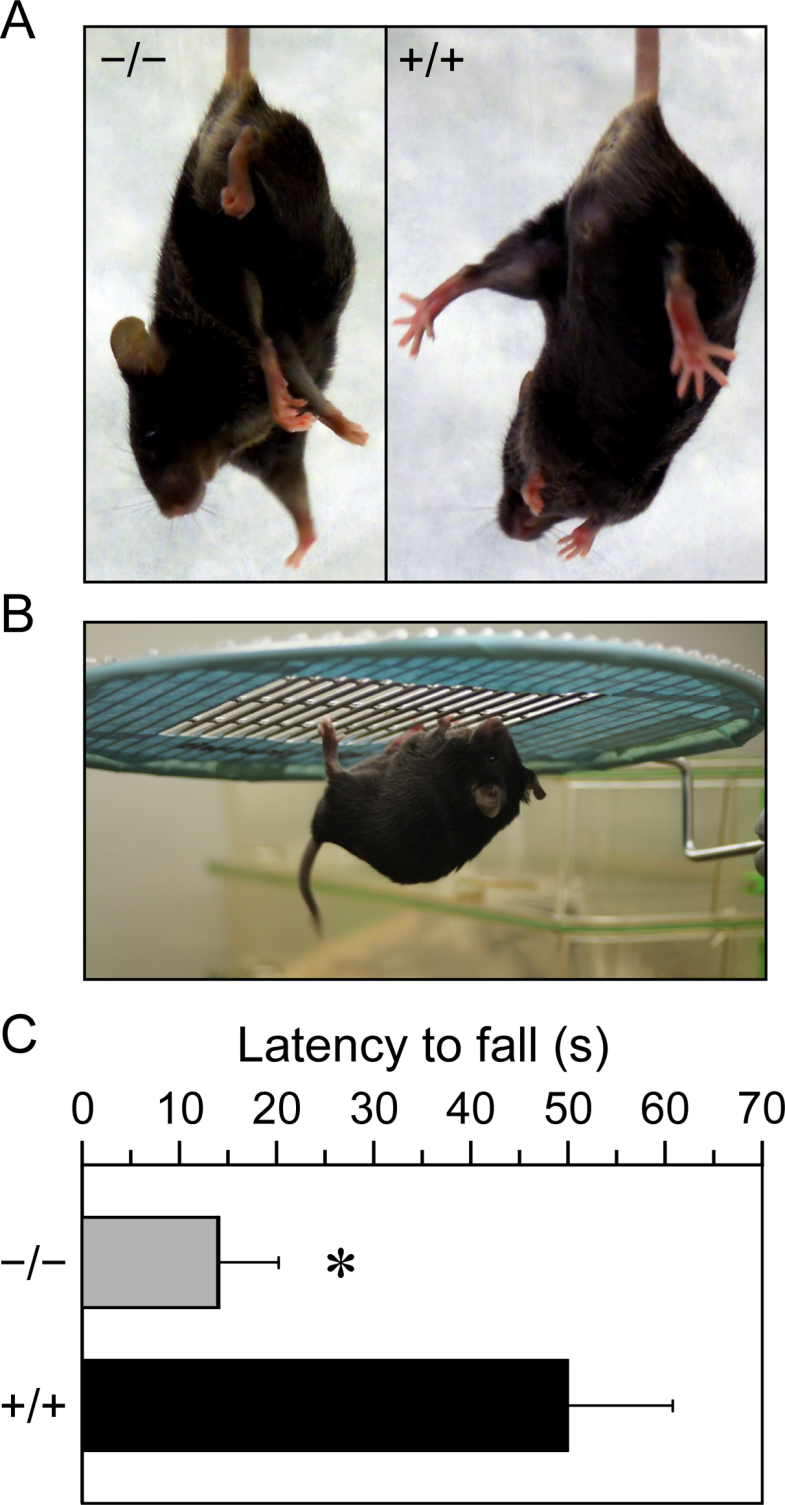
Neurologic assessment. **A,** Tail suspension test. *Kcc3*^−/−^ mice clasp both the forelimbs and hindlimbs upon suspension, whereas *Kcc3*^+/+^ mice extend them. **B and C,** Wire hang test. Latency to which mice fell from the horizontal grid was recorded with a chronometer. Data are shown as means ± SEM of 12 mice among 6 experiments using the longest time for each mouse. * indicates that the mean is significantly different statistically compared to wild-type littermates (*p* < 0.0001) based on a Wilcoxon rank-sum test.

To determine how allele expression in the genetic background exploited for this study compared with allele expression in the genetic backgrounds exploited for previous studies, our *Kcc3*^−/−^ mice were subjected to basic neurologic tests. As reported by other investigators and illustrated in Fig 8, the gait was characterized by dragging of the hindlimbs and tail suspension caused clasping of both the forelimbs and hindlimbs (see panel A where *Kcc3*^−/−^ mouse is on the left and *Kcc3*^+/+^ mouse is on the right). Motor abnormalities also appeared to compare with those observed in previous studies based on the wire hang test (panels B and C).

## Discussion

In this work, we have exploited a background-purified C57BL/6J mouse line to gain further insight regarding the role of KCC3 in the cardiovascular tissue and in the proximal nephron. As in other models of *Kcc3* inactivation [18–20], our model exhibited elevated MAP and several neurologic abnormalities. However, MAP was elevated mainly as a result of high DBP and this trait was also accompanied by isosmotic polyuria, cardiac hypertrophy and lower sensitivity of denervated aortas to PhE.

Although *Kcc3*^−/−^ mice were found to exhibit high MAPin a previous study, the effect of adrenergic agents on third generation saphenous arteries isolated from these animals was the same compared to WT mice, a finding that could be seen as inconsistent with a role for KCC3 in resistive vessels [20]. Given, however, that DBP values were not reported thereupon, one cannot exclude that VSMC tone was altered in precapillary arterioles. In the previous study, moreover, *Kcc3* inactivation also led saphenous arterial VSMCs to exhibit higher Cl^−^ concentrations.

It is noteworthy that in our own mouse model, SBP was virtually unaffected compared to WT littermates and that the aortic wall was both thinner and less sensitive to PhE. Given that the aortas were characterized in vitro while denervated, it is tempting to postulate that these abnormalities resulted from *Kcc3* inactivation in the vessel wall. Our findings also suggest that the aortas of *Kcc3*^−/−^_129/Sv×C57BL/6_ mice were more compliant than those of WT littermates although it was not possible to confirm this possibility further. In small rodents, unfortunately, pulse wave velocity measurements along the arterial tree are currently very challenging and not always easy to interpret.

It might appear paradoxical that resistive and conductive vessels would react differently to a given intervention or stimulus. Yet, this was shown to be the case in the rabbit pulmonary circulation where the effect of hypoxia on vessel tone was found to vary as a function of the VSMC subtypes present [29,30]. It was also shown to be the case in the cat cerebral circulation where pial arteries were found to dilate in response to norepinephrine [31]. Regarding our *Kcc3*^−/−^ mice more specifically, differences in the contractile responses of VSMCs along the arterial tree could be due to differences in the population of coexpressed ion transport systems.

If the absence of *Kcc3* in VSMCs caused DBP to increase and PhE-induced aortic contraction to decrease, what mechanisms could be involved? In animal models of *Kcc2* inactivation, it has been proposed that electroneutral accumulation of Cl^−^ in neurons predisposes to epilepsy by decreasing γ-aminobutyric acid receptor-mediated inward Cl^−^ currents secondarily [32]. Similar mechanisms could thus be at play in resistive vessels where inward Cl^−^ currents would also be expected to decrease in response to higher Cl^−^ concentrations associated with *Kcc3* inactivation [20]. Were K^+^ currents of greater amplitude than Cl^−^ currents in the vascular wall of conductive vessels, the absence of KCC3 could then exert opposite effects on VSMC tone. Currently, very little is known regarding the ion transport properties of VSMCs in large vessels.

In our study, the effect of *Kcc3* inactivation on DBP was robust, but not very strong. However, it was also associated with isosmotic polyuria that was not fully compensated for based on the amount of water ingested. Hence, some degree of volume contraction associated with *Kcc3* inactivation in the basolateral membrane of proximal nephrons could have prevented BP from increasing to higher levels [28]. Although aldosterone levels were not increased under a regular-salt diet, KCC3 was also found to be normally expressed in all layers of the adrenal gland (see Fig 6), implying that it could therefore play a role in hormonal synthesis or secretion. Downregulation of *Nkcc1* expression levels in a number of *Kcc3*^−/−^ tissues, as could be the case based on our preliminary qPCR studies, might have also blunted the increase in BP.

Our model of *Kcc3* inactivation was remarkable compared to the other models for exhibiting a slower heart rate and a 30%-increase in cardiac muscle mass due to hypertrophic changes. Both these phenotypic traits could have developed in response to chronically elevated DBP or neurogenic mechanisms. Another possibility, however, is that they could have also developed from inactivation of *Kcc3* in myocardial cells. This possibility is supported by the observations that an increase in *Nkcc1* activity, under which circumstances intracellular K^+^ and Cl^−^ concentrations are also higher, is often associated with higher rates of cell proliferation or growth *in vitro* [24,33,34]. A decrease in KCC3 activity would thus lead to similar responses.

Accessory findings in this work were that *Kcc3*^−/−^ mice exhibited lower weight at adult age and decreased fat deposition while chow intake was increased. They suggest that KCC3 could play a role in energy allocation as well as in the development of obesity or metabolic dysfunction leading to cardiovascular disorders. In particular, it would be of interest to determine whether energy expenditure is greater in *Kcc3*^−/−^ mice than in WT mice and whether neuronal, hormonal or a combination of mechanisms are involved.

Although differences were identified between our *Kcc3*^−/−^_C57BL/6J_ and previously characterized *Kcc3*^−/−^_129/Sv×C57BL/6_ mice, it remains uncertain to what extent phenotypes actually diverge. First, more subtle defects in one strain might have escaped detection. Second, certain defects might not have been reported or looked for in *Kcc3*^−/−^_129/Sv×C57BL/6_ mice. Third, the experiments carried out to characterize each strain were not the same. For instance, Rust et al. [20] used isolated saphenous arteries to study the contractile properties of arterial vessels while we used thoracic aortas.

In conclusion, we have found that *Kcc3* inactivation in VSMCs could be responsible for certain of the hemodynamic abnormalities observed in *Kcc3*^−/−^ mice and that it could exert differential effects in conductive and resistive vessels. We have also found that *Kcc3* was naturally expressed in the mouse heart and that its inactivation in the C57BL/6J background led to cardiac hypertrophy and negative chronotropism. Cl^−^ accumulation in the vessel wall and in the heart could thus play important pathogenic roles, but further investigations are required to confirm this hypothesis and understand the mechanisms involved. Our studies also highlight the importance of KCC3 in solute reabsorption by the proximal nephron.
